# No severe and global X chromosome inactivation in meiotic male germline of *Drosophila*

**DOI:** 10.1186/1741-7007-10-50

**Published:** 2012-06-12

**Authors:** Lyudmila M Mikhaylova, Dmitry I Nurminsky

**Affiliations:** 1 Department of Biochemistry and Molecular Biology, University of Maryland School of Medicine, Baltimore, MD, USA

## Abstract

This article is a response to Vibranovski *et al.*

See correspondence article http://www.biomedcentral.com/1741-7007/10/49 and the original research article http://www.biomedcentral.com/1741-7007/9/29

We have previously reported a high propensity of testis-expressed X-linked genes to activation in meiotic cells, a similarity in global gene expression between the X chromosome and autosomes in meiotic germline, and under-representation of various types of tissue-specific genes on the X chromosome. Based on our findings and a critical review of the current literature, we believe that there is no global and severe silencing of the X chromosome in the meiotic male germline of *Drosophila*. The term 'meiotic sex chromosome inactivation' (MSCI) therefore seems misleading when used to describe the minor underexpression of the X chromosome in the testis of *Drosophila*, because this term erroneously implies a profound and widespread silencing of the X-linked genes, by analogy to the well-studied MSCI system in mammals, and therefore distracts from identification and analysis of the real mechanisms that orchestrate gene expression and evolution in this species.

## Introduction

Although meiotic sex chromosome inactivation (MSCI) has been convincingly documented in mammals, and its presence in other taxa has often been inferred based on several lines of evidence [1], the widespread conservation of this phenomenon has been questioned by several recent studies, including ours, that have shown little evidence for MSCI in birds [2] and flies [3,4]. In mammals, MSCI presents as a global and severe silencing of the X chromosome, with over 80% of the X-linked genes being downregulated by fivefold or more [5]. By contrast, our analysis of the gene expression in the *Drosophila *male germline [3] has shown that in this species, the X-linked and autosomal genes have a similar propensity to activation in meiotic cells, and that the average gene expression in meiotic germline is similar between X chromosome and autosomes. Further, we presented evidence that the paucity of the X-linked testis-specific genes reflects a general under-representation of the tissue-specific genes on the X chromosome, which argues against the proposed role for MSCI in *Drosophila *genome evolution [6]. We concluded that a global meiotic sex-chromosome inactivation does not occur in *Drosophila*, and that other mechanisms such as specific chromatin modifications are more likely explanations for the paucity of the tissue-specific genes on the *Drosophila *X chromosome. However, these conclusions were questioned by Vibranovski *et al. *who suggested that 1) our choice of the normalizing 'housekeeping' control undermined our analysis of the propensity of the X-linked genes to activation in the meiotic germline, 2) small but significant underexpression of the X chromosome in testis tissue can be identified in our microarray data, and 3) under-representation of the tissue-specific genes on the X chromosome is not supported by statistical analysis. Further, Vibranovski *et al. *suggest that the current published research is still compatible with the MSCI hypothesis. In this paper, we address the theoretical and technical arguments presented by Vibranovski *et al. *We remain confident in our original research study, which does not support the global and profound meiotic silencing of the *Drosophila *X-linked genes that would be expected if MSCI were in operation in this species.

## High propensity of the testis-expressed X-linked genes to activation in meiotic cells

Previously, we used quantitative reverse transcriptase (qRT)-PCR to show that transcripts of the eight X-linked genes and the eighteen autosomal genes are similarly and dramatically upregulated in the testis of third instar *Drosophila *larvae as the primary spermatocytes increase in number and mature (days 5 to 7 of development) [3]. This finding is consistent with the induction of 94% of the 501 testis-expressed X-linked genes in primary spermatocytes that we reported in that study [3], based on analysis of the published *in situ *hybridization data [7], which showed at a cellular level that X-linked genes are commonly activated in the meiotic male germline. Vibranovski *et al. *question the extent of this upregulation, suggesting that expression of the *rp49*/*RpL32 *gene, which we used as a housekeeping control gene to normalize the qRT-PCR data, decreases approximately twofold during larval development. However, 1) this decrease in the *rp49 *expression in testis was an inference by Vibranovski *et al. *from the whole-animal gene expression data without any experimental support; 2) this putative decrease is small compared with the 10-fold upregulation of testis-expressed genes; 3) expression of both the X-linked and autosomal genes was uniformly normalized to the *rp49 *transcript, and therefore the similarity in their expression profiles is not affected by the variation in *rp49 *abundance; and 4) each analyzed gene set contained known spermatocyte-specific genes as internal controls, confirming that activation of other genes is concomitant with meiosis.

## Lack of severe global silencing of the X chromosome in testis development

We agree with Vibranovski *et al. *that there is a significant underexpression of the X chromosome at late stages of testis development, but this is small (7% or less). Vibranovski *et al. *suggest that the 'real' difference could be higher than that detected by our analysis basing these arguments on their critique of our microarray experiments. However, our studies are consistent with the work of others who have shown that there is no severe and global silencing of the X chromosome in *Drosophila *meiotic male germline. For example, Gupta *et al *[8] found no substantial difference between the X chromosome and autosomes in global gene expression in the testis. Further, Meiklejohn *et al. *recently showed that, regardless of the experimental model or of the high-throughput method used in previous studies [4,9,10], the global underexpression of the X chromosome compared with the autosomes in the *Drosophila *meiotic male germline was 33% at most. This is in striking contrast to the magnitude of changes in mammals expected from the high frequency and severity of the X-linked gene silencing in MSCI [5], and confirmed by the eightfold reduction in mammalian X-chromosome expression in the testis [11].

## Paucity of the tissue-specific gene expression on the *Drosophila *X chromosome

Previously, we reported that the genes whose expression was strongly biased towards the mapligian tubule, midgut, accessory gland, and salivary gland, are under-represented on the *Drosophila *X chromosome [3]. Vibranovski *et al. *question the statistical significance of these findings, and argue that about 60% of the mapligian tubule, midgut, accessory gland, and salivary gland-biased genes in our datasets also have low-level expression in the testis and ovaryI, and that the expression of these genes varies between these gonad tissues. They define these genes as 'testis-biased' or 'ovary-biased', remove them from the datasets as potentially confounding factors, and report a lack of significant under-representation of the remaining genes. However, there are some fundamental problems with such an analysis. For example, a gene is usually defined as testis-biased if its expression is higher in the testis than in a number of other tissues, not just in one arbitrarily chosen tissue (in this case the ovary). The same is true for the ovary-biased genes. Defining the genes with low-level expression in testis or ovary as testis-biased or ovary-biased seems to be misleading, because this gives a false impression of their strongly suggested function in germline. There is therefore no strong justification for exclusion of these genes from the datasets and, importantly, such exclusion leads to non-specific loss of the significance of analysis (we confirmed that statistical significance is lost after random eliminations of 60% of the genes from our datasets). Further, we performed an additional statistical analysis of our published data, and found that under-representation of the midgut, accessory gland, and salivary gland-biased genes on the X chromosome compared with the autosomes is significant (*P *≤ 0.05), and that under-representation of mapligian tubule-biased genes has a lower yet substantial support of significance (*P *≤ 0.1) (two-proportion *z*-test). Thus, our hypothesis that the X chromosome provides an inferior environment for diverse types of tissue-specialized genes gains further support.

## Discussion

Three lines of experimental evidence are outlined in the article of Vibranovski *et al. *as supportive for MSCI in *Drosophila*. First, the insertions of transgenes driven by a testis-specific *ocnus *promoter show significantly decreased expression when integrated into the X chromosome, compared with the autosomes [12,13]. Second, the analysis of testis in the *bam *mutant, which lacks meiotic cells, showed higher X-chromosome expression compared with wild-type testis [4,9]. Third, an earlier study showed statistical evidence for downregulation of the X chromosome during meiosis, including a very small but significant excess of the downregulated X-linked genes [10]. In addition, the exodus of the testis-specific retroposed genes from the X chromosome can be viewed as indirect evidence for global meiotic X-chromosome inactivation [6].

However, these lines of evidence have recently been refuted. Silencing of the X-linked *ocnus*-driven transgenes in male germline has been shown to be established in mitotic rather than in meiotic cells, and a significant reduction in global X chromosome expression compared with the autosomes is already seen in the undifferentiated mitotic germline present in the *bam *mutant testis [4]. Therefore, the mechanism(s) responsible for the lower expression of the X-linked genes in the male germline are not tied to meiosis. Further, when the expression data from the previous study [8], showing downregulation of the X chromosome in male meiotic cells, was re-analyzed using a conventional statistical approach, there was no significant evidence for the excess of the X-linked genes downregulated in meiotic cells [3]. Underexpression of X chromosome in male germline has been estimated to be less than 33% [3,4,9,10], which is several times smaller than the difference between the X chromosomes and autosomes in mammalian MSCI [11], and is almost identical to the effect of lack of dosage compensation expected in male meiosis [4,14]. Finally, a recent study showed that a preferred autosomal integration may be an intrinsic property of retroposing genes, regardless of their expression pattern [15], and at present, there is no published evidence indicating otherwise.

## Conclusions

Our original research article and the work of others indicate that there is no evidence for global and severe X chromosome inactivation in *Drosophila *male germline. Although there is a slight underexpression of X chromosome in testis, the use of term 'MSCI' in referring to this effect seems to be misleading, and distracts from the identification and analysis of underlying mechanisms.

## Authors' contributions

LMM and DIN analyzed the data and wrote the manuscript. All authors have read and approved the final manuscript.
